# Work–family and family–work conflict and stress in times of COVID-19

**DOI:** 10.3389/fpsyg.2022.951149

**Published:** 2022-10-11

**Authors:** Natasha Saman Elahi, Ghulam Abid, Francoise Contreras, Ignacio Aldeanueva Fernández

**Affiliations:** ^1^Superior College Lahore, Lahore, Pakistan; ^2^Department of Business Studies, Kinnaird College for Women University, Lahore, Pakistan; ^3^School of Management and Business, Universidad del Rosario, Bogotá, Colombia; ^4^Department of Economics and Business Administration, Universidad de Málaga, Málaga, Spain

**Keywords:** work–family conflict, family–work conflict, stress, COVID-19 pandemic, balance work-family, spillover impact

## Abstract

This study aims to investigate the spillover impact of work-family/family–work conflict and stress on five major industrial sectors (education, textile, hospitals, banks, and retail stores), during the first wave of Covid-19. The purpose of this cross-sectional study is twofold; firstly, to test a hypothesized model where work-family/family-work conflicts are related to stress and where stress could exert a mediating role in such relationships. Secondly, we seek to explore the presence of these conflicts and stress in each of the five major industrial sectors and evaluate if there are significant differences between them, identifying the sociodemographic characteristics associated. Two questionnaires were applied to 748 employees from the selected industries. According to our results, stress predicts both types of conflict and also exerts a mediator role. It was primarily found that the five sectors are significantly different regarding the work-family/family-work conflicts and stress. Findings and implications are discussed.

## Introduction

The world has been committed to implementing the 2030 agenda for sustainable development; however, the unforeseen conditions brought on by Covid-19 at the start of 2020 are badly influencing this promise and undermining the universal path towards sustainability by slackening down the process (Shulla et al., 2021). The Covid-19 pandemic has had an unpleasant, undesirable, unclear, difficult to measure, and long-lasting significant influence on the Sustainable Development Goals (SDGs) approved by the United Nations in 2015. Out of those 17 interlinked global goals, the 3rd goal of the SDGs is oriented towards ensuring healthy lives and promoting the well-being of all age groups, including working employees. This specific goal asserts that every individual must be in good health and mentally and physically fit (well-being) in order to endorse social sustainability and development in the world for the advancement of future generations and the global community (Abid et al., 2020). Nevertheless, the Covid-19 pandemic has impacted employees around the globe, changing the way in which people work and live (Contreras et al., 2020; Rana et al., 2021). Therefore, as a drastic measure to shorten the curve of this mass infection and continue operations, public and private organizations have had to adopt technology in their processes and their employees had to suddenly become teleworkers (Contreras et al., 2020). Telework has proven beneficial to its customers, allowing them to balance their professional and personal lives (Vilhelmson and Thulin, 2016). However, Felstead and Henseke's (2017) study has demonstrated that teleworking can adversely affect work-life balance. Under normal circumstances, balancing work and family obligations is difficult for working people who frequently report family and work conflicts (Andrade and Petiz Lousã, 2021). In the COVID-19 epidemic, the boundary between work and personal life presented additional problems, potentially resulting in conflict between work and family or family and work due to changes in the workplace, home environment, and social relationships (Yildiz et al., 2021). This situation can harm the work-life interface, mental well-being, and stress. The term “stress” is the unpleasant emotional response that humans may develop in or outside the workplace when they perceive that they do not have an adequate response to a perceived threat, resulting in frustration and anxiety (Seaward, 2019). In the view of Kihara and Mugambi (2018), stress can be caused by a variety of factors such as working conditions that are not conducive to productivity and health, work overload, the inability to deal with work demands or express grievances because of a dread of being laid off, lack of engagement and work-life balance (Soomro et al., 2018; Seaward, 2019; Dodanwala et al., 2022). Many academics focus on the link between extreme environmental or working conditions and rising employee stress because of the current COVID-19 pandemic crisis, which causes workplace instability. However, the full impact of the work-family/family-work on employee stress and stress effect on family/work conflict in the COVID-19 pandemic is still unclear, which provided a new milieu for understanding this area. Understanding the work-family/family-work conflicts and the stress employees have been experiencing in the COVID-19 pandemic is crucial. Therefore, our study explores this effect to void this gap in the Asian context.

Moreover, Covid-19 is significantly impacting the work and life of many people around the world. It is causing fear, stress, loneliness, panic, depression, fear of worthlessness, overwhelming work pressure, burnout, isolation, uncertainty, anxiety, and even substance abuse in some people (Dubey et al., 2020; Rana et al., 2021) and family/work conflict (Dodanwala et al., 2022). Likewise, although it can be assumed that these conditions can be experienced by employees from all sectors (Cui and Li, 2021; Yildiz et al., 2021), we need to understand if there are higher risk groups in the major industries such as health care, banking, education, textiles, and retail sectors. This is because limited research has explored the presence of these conflicts and stress in each of the five major industrial sectors in the Asian organizational setting. In order to void the gap, this study addresses these issues by analyzing (1) the impact of work to family conflict on stress and (2) the impact of stress on family to work conflict during the global pandemic situation. (3) We also investigate the reciprocity of these relationships in another study with an independent sample. Finally, we contextually explore both the phenomena of work–family conflict dimensions and stress in order to identify the severity of their prevalence in different sectors.

## Literature review and hypotheses development

### Work–family conflict and family–work conflict

In pandemic times, the boundaries between work and family are increasingly unclear, creating conflicts between the employees’ work and family dimensions (Novitasari et al., 2020; Phillips et al., 2020; Vaziri et al., 2020). However, work and family conflict is not exclusive to pandemic situations (Powell, 2020). This conflict already existed before the pandemic and was rapidly growing due to the socio-economic changes that the world has been experiencing, it was only exacerbated by the presence of Covid-19 (Poggesi et al., 2019), affecting both developing and developed countries (Soomro et al., 2018). Although work and family conflict was defined more than 30 years ago, this issue recently gained more relevance considering that the number of single parents, dual-earner couples, and households living with aging parents has increased in the last years (Bennett et al., 2017).

Initially, work and family conflict was defined as “a form of inter-role conflict in which the role pressures from the work and family domains are mutually incompatible in some respect” (Greenhaus and Beutell, 1985, p: 77). Subsequently, empirical evidence established that work and family conflict should be seen as a bidirectional concept (Frone et al., 1992), that is to say, family–work conflict and work–family conflict (Mäkelä and Suutari, 2011). Netemeyer et al. (1996, p: 401) defined work–family conflict as “a form of inter role conflict in which the general demands of time, devoted to, and strain created by the job interfere with performing family-related responsibilities,” while family–work conflict is defined as “a form of inter role conflict in which the general demands of time devoted to and strain created by the family interfere with performing work-related responsibilities.” The two notions are adequately dissimilar in scope and nature to necessitate their investigation (Mesmer-Magnus and Viswesvaran, 2005). Byron (2005: metanalysis) found that the work-family/family–work conflict has distinct antecedents and attitudes (non-work and related variables, demographics). The findings of previous research have confirmed the distinctness of the two concepts. Thus, the current studies explore and consider both sides of the conflict (Soomro et al., 2018).

Work and family spheres involve different responsibilities, which can become a permanent challenge for the employees and reinforce the inter-role conflict within the individual (Anatan, 2013; Jamadin et al., 2015; Hasan et al., 2020). Sometimes, the incompatible demands between work and family produce personal pressure on employees (stress) that could be the source of several health problems. Thus, reducing work-family/family–work conflict is crucial to protect the workers’ health, which is something that needs to be supported by human resources management in companies if they wish to improve employees’ psychological well-being and organizational performance (Arslaner and Boylu, 2017; Lee, 2018). In times of Covid-19, more than ever the work-family and family-work conflicts are important issues that need to be studied to improve the employees’ well-being and, as a consequence, the organizational outcomes, given that employees need to achieve an optimal balance between the spheres of work and family without being submitted to the dilemma of giving priority to work or family. When employees feel social support to deal with the work and family responsibilities, it can buffer the daily work–family conflict (Pluut et al., 2018). The relevance and benefits of social support are clear and the organizational support can be the greatest source of support in this regard (French et al., 2018).

Regarding the demographic features, a gendered approach deserves special attention since family and work conflicts affect more women than men due to the gender roles historically established (McElwain et al., 2005). For this reason, gender is today a relevant topic in the literature about work and family conflict and it has been studied in different industrial sectors such as banking institutions (Aboobaker and Edward, 2020), universities (Calvo et al., 2012; Hong et al., 2021), health (Yildiz et al., 2021), public service, finance, education, and non-government organizations (Drummond et al., 2017), manufacturing (Cui and Li, 2021) and service (Haines et al., 2019), and manufacturing, retailing, and finance (Kim and Gong, 2017).

Other demographic characteristics that have been shown as being related to the conflicts between work and family are age, education, working hours and family hours, income, number of children, employment (Abeysekera and Gahan, 2019), educational qualification, total years of work experience, position in the organization (Aboobaker and Edward, 2020), presence of children living at home, level of occupation, living with a partner, marital status, children’s age (Calvo et al., 2012), race (Cloninger et al., 2015), number of dependents currently living with respondents (Drummond et al., 2017), working full or part-time, and number of the children at home (Hagqvist et al., 2017; Haines et al., 2019).

### Work–family/family–work conflict and stress

Work–family conflict is one of the most studied organizational behavior topics (Netemeyer et al., 1996). Work and family are integral components of the life of any working person. However, work–family conflicts can arise when roles are incompatible. This work–family conflict/family-to-work conflict leads to several negative consequences, such as job dissatisfaction (Lu et al., 2017), lower work-life balance, job satisfaction (Talukder, 2019), job performance (Soomro et al., 2018), work engagement (Lyu and Fan, 2020) and higher emotional exhaustion (Wang et al., 2012), emotional intelligence and self-efficacy (Zeb et al., 2021).

Like outcome scholars identified that antecedents of work–family conflict have been associated with three different categories: work, non-work (i.e., family), and health-related consequences (Amstad et al., 2011). Andrade and Petiz Lousã (2021) revealed that low job autonomy, role overload, and after-hour work-related technology predicted the work family conflict in Covid-19. Michel et al. (2011) found that work role stressors, work role involvement, personality, work characteristics, social support at work (Talukder, 2019), proactive behavior and workplace anxiety are the predictors of work–family conflict (Cui and Li, 2021). Whereas family role stressors, family characteristics, family social support, and personality are the predictors of family–work conflict. Freire and Bettencourt (2020) found that ethical leadership is the predictor of work family conflict. Hargis et al. (2011) asserted that job stressors and negative affectivity are more crucial predictors of work–family conflict. It is worth noting that a stressor is any perceived feature of the setting that threatens, harms, and/or challenges the employees (Latack, 1986). Rees (1997, p: 35) defined job stress as “the inability to cope with the pressures in a job.” Stress due to work–family conflict is not only uncomfortable and undesirable but also of a permanent nature.

Mansour and Tremblay (2018) recall the two approaches to stress at work, i.e., transactional approach which highlights the stress development between people and the work environment; and the interactionist approach which contemplates stress as a consequence of an interface between people and the environment. It is suggested that the extent of stress an individual experiences at the workplace is most likely the outcome of the interface of various dynamics like the job type, the presence of stressors, and the extent of support obtained from both home and work (Johnson et al., 2005). Additionally, individuals working in the same profession may sometimes experience dissimilar stress levels because of the interaction of their personality types. With the increase in job stress, there is an increasing emphasis placed on the studies related to work–family conflict (Zhang and Liu, 2011).

Overall, stress is linked with work–family conflict and family–work conflict. In literary reviews, we have heterogeneous evidence of this; e.g. Indonesian auditors (Amiruddin, 2019); Canadian workers in different sectors as government, private for-profit, non-profit organizations, self-employed (Badawy and Schieman, 2020); American (Griffin and Sun, 2018) and Indian (Lambert et al., 2017) police officers; Chinese bank employees (Kan and Yu, 2016); Chinese prison staff (Liu et al., 2017); Turkish primary teachers (Nart and Batur, 2014); Belgian working mothers (Vercruyssen and Van de Putte, 2013). As Tziner and Sharoni (2014) asserted, culture influences differently the way stress is perceived and managed at the workplace and how the family copes with work-related pressures. On the other hand, work–family conflict is a substantial factor that contributes to stress, and this connection has been thoroughly researched (Vickovic and Morrow, 2020; Dodanwala et al., 2022).

According to the above, we posit the following hypotheses:

*H*1. Work–family conflict influences stress positively and significantly.

*H*2. Stress influences family–work conflict positively and significantly.

*H*4. Family–work conflict influences stress positively and significantly.

*H*5. Stress influences work–family conflict positively and significantly.

According to the above, we posit the following hypotheses:

### Stress as mediator

Stress as the specific mediator between these two types of conflicts in times of pandemic is not yet present in the literature. Nevertheless, there are some evidences of stress as a mediator in the work-family context. For example, it is already known that the basic elements of burnout, i.e., cynicism and emotional exhaustion, are considered a response to permanent work stress (Rubio et al., 2015). Dodanwala and San Santoso (2021) found that job stress mediates the job satisfaction and turnover intention association. It has also been established that burnout mediated the association between bullying and work–family conflict (Raja et al., 2018). Irawanto et al. (2021) found the mediating role of work stress between working from home and job satisfaction. Ismail and Gali (2017) also explore the mediating role of stress at work between work–family conflict and satisfaction with performance appraisal.

According to the above, we posit the following hypotheses:

*H*3. Stress mediates the association between work–family conflict and family–work conflict.

*H*6. Stress mediates the association between family–work conflict and work–family conflict.

**Figure 1:**
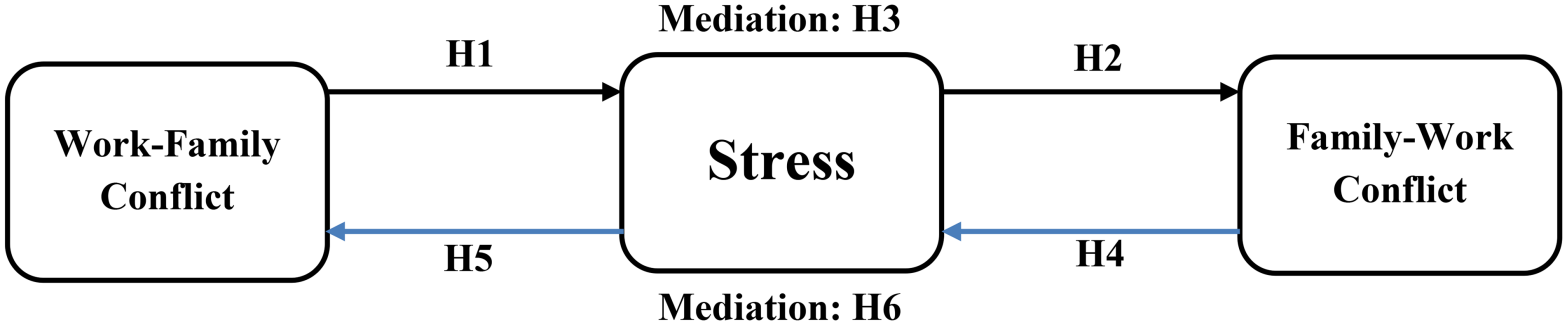
Hypothesized research model.

The Hypothesized research model is presented in Figure 1.

## Materials and methods

### Sample and procedure

This research used a cross-sectional survey study designed for data gathering. Since this research is cross-sectional, the data was collected from participants at one point in time in both the study 1 and the study 2. The survey questionnaires were distributed during the first wave of the Covid-19 period using a purposive sampling technique across one of the major metropolitan city of Pakistan. The questionnaires were distributed personally within the allocated time to the respondents who came to the workplace. During the data collection process, we ensured (a) the confidentiality of the respondents, (b) voluntary participation, and (c) that the data was gathered by the researcher without involving the top authority (Iqbal et al., 2020). Furthermore, the participants had to be permanently employed full time by the five targeted sectors, i.e., education, textiles, hospitals, banks, and retail stores. Moreover, the questionnaires were administered in English and the data collection procedure for both the studies was the same.

In the study 1, a total of 600 questionnaires were distributed equally among education, textile, and hospitals. The total of valid responses was of 400. Of these responses, 52.3% were male and 49.3% were single. Most of the participant’s age (35.5%) ranged from 30 to 39 years. The majority of the participants (51.3%) had a university degree; some of the participants (17%) have tenure above 20 years, while the overall experience of the majority of participants (36%) ranged from 0 to 10 years.

Regarding the sample from study 2, a total of 400 questionnaires were distributed equally among banks and retail stores. The total of valid responses was 348, most of them were male (86.5%) and married (50.9%) with ages that ranged from 20 to 29 (42.8%). The majority of respondents (54.9%) are graduated with tenure ranged from 6 to 10 years (35.6%), while the overall work experience of the majority of participants ranged from 0 to 10 years (42.8%).

### Measures

#### Family-to-work conflict

We used a five-item scale developed by Anderson et al. (2002) to measure perceptions of the extent to which one’s family interferes with one’s work. A sample item from the scale was “In the past 3 months, how often has your family or personal life kept you from getting work done on time at your job?”

#### Work-to-family conflict

We used a five-item scale developed by Anderson et al. (2002) to measure perceptions of the extent to which one’s work interfered with one’s family. A sample item from the scale was “In the past 3 months, how often have you not had enough time for yourself because of your job?”

#### Stress

We used a seven-item scale developed by Anderson et al. (2002) as an indicator to measure the stress in the workplace. A sample item from the scale was “During the past 3 months, how often have you felt emotionally drained from your work?”

We used a 5-point Likert type scale from (1) *never* to (5) *always* for all the three constructs. We coded the items such that higher numbers represent more frequent experiences of family interference with work, work interfering with family and stress.

## Results

### Outliers and data normality analysis

To check the outliers in Study 1 and 2, we utilize Mahalanobis Distance (MD) and developed the MATRIX cumulative distribution. The MD values ranged as [Study 1: (0.00178 to 13.80) and Study 2: (0.00202 to 13.85929)]. Furthermore, MATRIX cumulative distribution values ranged as [Study 1: (0.00101–0.99911) and Study 2: (0.00125 to 0.99899)]. These results indicated the absence of outliers in the data sets.

We applied Kurtosis and Skewness to check the data normality. Kurtosis measures the tail extremity reflecting the presence of outliers, whereas Skewness measures the direction and degree of asymmetry. The responses for all the items were normally distributed with Kurtosis ranging between +3 and − 3 [Study 1: (−0.037 to −0.311) SE 0.243 and Study 2: (−0.071 to −0.563) SE 0.261] and Skewness ranging between +1 and − 1 [Study 1: (−0.191 to 0.565) SE 0.122 and Study 2: (−0.022 to 0.542) SE 0.131] (Hair et al., 2014).

### Convergent and discriminant validity

First, CFA (confirmatory factor analysis) was conducted for the purpose of determining instrument validity by following Fornell and Larcker’s (1981) validity assessment criteria on the combined data set of both the studies. Before assessing convergent and discriminant validity through CFA, model fit indices as well as alternate models were evaluated for our measurement model. Initially, a full measurement model that consisted of three factors was examined. For this purpose, we drew all our items, i.e., 5-items of work–family conflict, 7-items of stress, and 5-items of family–work conflict, in AMOS (24 version), then relevant items were connected and permitted to correlate liberally onto their respective factors. The three-factor model fit incidences such as GFI (0.93), TLI (0.91), CFI (0.91) and RMSEA (0.07) met the acceptable criteria (Hu and Bentler, 1999; Table 1).

**Table 1:** Comparison of measurement and alternative models fit indices.

Models	Combination	CMIN	DF	CMIN/DF	GFI	IFI	CFI	RSMEA
Measurement model	Work–family conflict, stress, family–work conflict	469.37	100	4.69	0.93	0.91	0.91	0.07
2 Factor model	Work–family conflict and family–work conflict into one factor	3179.8	118	18.4	0.69	0.53	0.53	0.15
1-Factor model	All factors/items combined into one	2672.9	119	22.4	0.64	0.42	0.42	0.17

Secondly, Fornell and Larcker’s (1981) recommendations were used for the assessment of the validity (convergent and discriminant) of measures. Results illustrated that Composite Reliability values of all constructs were surpassing the satisfactory limit >0.7, which confirmed the convergent validity. Likewise, the square root value of AVE for, i.e., family- work conflict, work–family conflict, and stress (0.73, 0.67, and 0.58) were greater than the intra-construct correlation, respectively. Hence, the criteria for discriminant validity were achieved (Gaskin, 2016; Table 2).

**Table 2:** Construct validity.

Variables	Convergent Validity	Discriminant Validity		
	CR	1	2	3
1. Family–Work Conflict	0.86	**0.76**		
2. Work–Family Conflict	0.80	0.12	**0.67**	
3. Stress	0.75	0.30	0.48	**0.58**

Bold values in diagonal represent the squared root estimate of AVE.

### Descriptive statistic and correlation matrix

The mean, standard deviation, and correlation among the control variables and study variables relating to Study 1 and Study 2 are presented in Table 3. Correlation coefficients provide the preliminary support for the stipulated hypothetical association among variables. In study 1, results demonstrate that work–family conflict is positively and significantly related to stress (*r* = 0.28, *p* < 0.01) and stress is positively and significantly related to family–work conflict (*r* = 0.14, *p* < 0.01). Moreover, correlation analysis reveals that gender is positively related to work–family conflict (*r* = 0.21, *p* < 0.01) and is negatively related to family–work conflict (*r* = −0.11, *p* < 0.05). Likewise, marital status is positively related work–family conflict (*r* = 0.12, *p* < 0.05) and negatively related to family–work conflict (*r* = −0.11, *p* < 0.01). Employee age, education, tenure, and experience are not associated with study variables in study 1.

**Table 3:** Means, standard deviations, and intercorrelations.

Study 1											
Variables	Mean	SD	1	2	3	4	5	6	7	8	9
Gender	1.48	0.5	1								
Marital status	1.48	0.5	0	1							
Age	3.08	0.99	−0.29^**^	0.23^**^	1						
Education	3.93	0.88	0.01	0.00	0.01	1					
Tenure	3.17	1.32	−0.02	0.01	0.24^**^	−0.01	1				
Experience	2.22	1.30	0.00	−0.05	0.06	−0.07	0.15^**^	1			
Work -family conflict	2.98	0.79	0.21^**^	0.12^*^	−0.06	0.09	0.00	0.00	**0.76**		
Stress	2.54	0.71	0.03	−0.03	−0.07	−0.03	0.01	−0.06	0.28^**^	**0.78**	
Family–work conflict	2.49	0.89	−0.01^*^	−0.21^**^	−0.02	−0.05	0.08	0.15^**^	0.06	0.14^**^	**0.80**
Study 2											
Variables	Mean	SD	1	2	3	4	5	6	7	8	9
Gender	1.14	0.34	-								
Marital status	1.51	0.5	−0.04	-							
Age	2.66	0.89	−0.12^*^	0.36^**^	-						
Education	3.35	1.19	0.02	0.14^**^	0.13^*^	-					
Tenure	2.89	1.36	−0.06	0.14^**^	0.17^**^	0.14^**^	-				
Experience	2.23	1.38	−0.03	0.14^**^	−0.06	−0.02	0.00	-			
Family–work conflict	2.43	0.93	0.11^*^	−0.01	−0.04	0.09	0.00	0.16^**^	**0.82**		
Stress	2.7	0.74	0.13^*^	−0.09	−0.13^*^	−0.12^*^	0.00	0.16^**^	0.45^**^	**0.73**	
Work–family conflict	3.15	0.93	−0.11^*^	−0.02^**^	−0.12^*^	−0.28^**^	−0.04	0.12^*^	0.16^**^	0.37^**^	**0.85**

Values in diagonal and bold represent the Cronbach’s alpha.

* *p* < 0.05 level;

** *p* < 0.01 level.

The correlation analysis from study 2 reveals that family–work conflict is positively (*r* = 0.45, *p* < 0.01) and significantly related to stress, which in turn is positively and significantly related to work–family conflict (*r* = 0.37, *p* < 0.01). Furthermore, results demonstrate that gender is positively related to family–work conflict (*r* = 0.11, *p* < 0.05) and stress (*r* = 0.13, *p* < 0.05), while it is negatively related to work–family conflict (*r* = −0.11, *p* < 0.05). Likewise, marital status is negatively related work–family conflict (*r* = −0.21, *p* < 0.05). Furthermore, age is negatively associated with stress (*r* = −0.13, *p* < 0.05) and work–family conflict (*r* = −0.12, *p* < 0.05), education is also negatively associated with stress (*r* = 0.12, *p* < 0.05) and work–family conflict (*r* = −0.28, *p* < 0.05). In addition, results indicate that experience is positively related with family–work conflict, stress (*r* = 0.16, *p* < 0.01) and work- family conflict (*r* = 0.12, *p* < 0.05).

### Hypotheses testing (Study 1)

In the present study, the path analysis approach (Amos 24) was used to test the direct and indirect path coefficients of the proposed holistic model in Study 1 and Study 2. Firstly, we analyzed whether the influence of work–family conflict on family–work conflict could be predicted with the stress in study 1. The outcome of path analysis shows that work–family conflict is positively and significantly related to stress (*β* = 0.25 *p* < 0.001), supporting H1. Results also show that stress is positively related to the family–work conflict (*β* = 0.18, *p* < 0.001), supporting H2. These outcomes provide support for mediation analysis. The results of the mediation model specified that stress mediates the association between work–family conflict and family–work conflict in a positive and significant way (*β* = 0.05, S.E = 0.02, *p* < 0.01, LB = 0.01, UB = 0.10), supporting H3 (Table 4).

**Table 4:** Work to family conflict, stress, and family to work conflict.

	Direct Effect				
Paths	β	S.E	CR		*p* Value
Work–family conflict→stress	0.25	0.04	5.95		^***^
Stress →Family-work conflict	0.18	0.06	2.93		0.00
	Indirect Effect				
	β	S.E	p	LB	UB
Work-family conflict→stress→family-work conflict	0.05	0.02	0.01	0.01	0.10

Significant at ***p < 0.01 level. N = 400, β = Unstandardized Coefficients; S.E = Standard Error; LB = Lower Bond; UB = Upper Bond

### Hypotheses testing (Study 2)

Furthermore, in study 2 we analyzed whether the influence of family–work conflict on work–family conflict could be predicted by stress. The outcomes show that the direct effect of family–work conflict on stress is positive and significant (*β* = 0.36, *p* < 0.001), supporting H4. Results also demonstrate that stress influences the work–family conflict in a positive and significant manner (*β* = 0.46, *p* < 0.001), supporting H5. These outcomes provide the support for mediation. In line with H6, results reveal that family–work conflict indirectly influences work–family conflict through the incorporation of stress as a mediator (*β* = 0.16, S.E = 0.03, *p* < 0.001, LB = 0.10, UB = 0.24; Table 5).

**Table 5:** Family to work conflict, stress, and work to family conflict.

	Direct Effect				
Paths	β	S.E	CR		*p* Value
Family–work conflict→stress	0.36	0.03	9.37		^***^
Stress→work-family conflict	0.46	0.06	7.47		^***^
	Indirect Effect				
	β	**S.E**	*p*	**LB**	**UB**
Family–work conflict→stress→work–family conflict	0.16	0.03	0.00	0.10	0.24

Significant at ***p < 0.01 level. N = 348, β = Unstandardized Coefficients; S.E = Standard Error; LB = Lower Bond; UB = Upper Bond

### Contextual differences among five major sectors

The aim of this first part of the analysis was to determine whether there was any significant difference among various groups (i.e., education, textiles, hospitals, banks, and retail stores) on the basis of work-to-family conflict, stress, and family-to-work conflict. We used the non-parametric test to see this difference between different groups, as our data did not fulfill parametric test assumptions (One-way ANOVA). For example, the Kolmogorov–Smirnov Z test shows that data did not follow the normal distribution for work-to-family conflict, stress, and family-to-work conflict, i.e., *p* < 0.001, all the three variables are less than 0.05. The findings also indicate that the mean value for work–family conflict is 3.06 ± 0.86, stress 2.61 ± 0.73 and family-to-work conflict is 2.46 ± 0.91 (Table 6).

**Table 6:** One-sample Kolmogorov–Smirnov test.

		Work-to-family conflict	Family-to-work conflict	Stress
N		748	748	748
Normal Parameters^a^^,^^b^	Mean	3.06	2.46	2.61
	Std. Deviation	0.86	0.91	0.73
Most Extreme Differences	Absolute	0.11	0.12	0.12
	Positive	0.06	0.12	0.08
	Negative	−0.11	−0.06	−0.12
Test Statistic		0.11	0.12	0.12
Asymp. Sig. (2-tailed)		0.00^c^	0.00^c^	0.00^c^

a Test distribution is Normal.

b Calculated from data.

c Lilliefors Significance Correction.

Also, the prerequisite for the parametric test such as equality of variance was not fulfilled since the value of p of the variance test homogeneity is >0.05 (Table 7). Thus, we have not used (parametric test: ANOVA) to figure out the difference between five groups on the basis of work-to-family conflict, stress, and family-to-work conflict. The above-mentioned findings of all prerequisites led us to use non-parametric tests, i.e., Kruskal-Wallis test.

**Table 7:** Test of homogeneity of variances.

Variables	Levene Statistic	df1	df2	Sig.
Work-to-family conflict	35.40	4	743	0.00
Family-to-work conflict	3.62	4	743	0.006
Stress	31.77	4	743	0.00

The results of Kruskal-Wallis test show that all the five groups are significantly different on the basis of work-to-family conflict, i.e., Chi-Square = 157.04, *p* < 0.001. The results also showed that retail store employees experienced higher work-to-family conflict (M = 514.69), followed by educational (M = 407.52), textile (M = 255.62), hospitals (M = 31.57), and banks (M = 262.75) respectively.

In addition, all the five groups are significantly different on the basis of stress, i.e., Chi-Square = 60.86, *p* < 0.001. Results indicate that employees in hospitals experience higher stress with a higher mean (M) of 428.05, followed by other groups, i.e., retail stores (M = 425.55), banks (M = 375.22), education (M = 290.98), and textile (M = 273.55) respectively.

Finally, it was also found that all the five groups are significantly different on the basis of family-to-work conflict, i.e., Chi-Square = 44.13, *p* < 0.001. Furthermore, the textile group faces the higher level of family-to-work conflict with a mean of M = 485.22, followed by other groups, i.e., banks (M = 427.78), hospitals (M = 370.94), retail stores (M = 318.72), and education (M = 246.65; Tables 8, 9).

**Table 8:** Test of statistics.

	Work-to-family conflict	Stress	Family-to-work conflict
Chi-Square	157.04	60.86	44.13
Df	4	4	4
Asymp. Sig.	0.000	0.000	0.000

a. Kruskal Wallis Testb. Grouping Variable: Sector

**Table 9:** Mean rank of groups.

Sector		N	Mean Rank
Work-to-family conflict	Education	150	407.52
	Textile	72	255.62
	Hospitals	178	331.57
	Banks	149	262.75
	Retail Stores	199	514.69
	**Total**	**748**	
Stress	Education	150	290.98
	Textile	72	273.55
	Hospitals	178	428.05
	Banks	149	375.22
	Retail Stores	199	425.55
	**Total**	**748**	
Family-to-work conflict	Education	150	346.65
	Textile	72	485.22
	Hospitals	178	370.94
	Banks	149	427.78
	Retail Stores	199	318.72
	**Total**	**748**	

## Discussion

In any occupation, it is challenging to differentiate work life with family life. Participants in this research work in an atmosphere that includes round-the-clock supervision, instability, long working hours, pressures to complete tasks on schedule and accurately, shift and night work. They are also obligated to play many roles at home (e.g., wife, spouse, father, daughter and son) and at work (employee), and yet they often strain to manage these responsibilities. As a result, participants in this research can experience conflicts in balancing work and family responsibilities, such as feeling blemished because they are not available to spend quality time with their families due to time spent managing job expectations, and vice versa. These situations lead to negative outcomes, such as stress. In order to inspect work–family conflict in the five sectors, the first objective of our study is to test a hypothesized model where we evaluate whether work–family conflict and family–work conflict are associated with stress. Also, stress could mediate between these two types of conflict.

Our study confirms the connection of work–family conflict with stress. This result is consistent with previous studies (Jamadin et al., 2015; Kusumanegara et al., 2018; Vickovic and Morrow, 2020) that found that work–family conflict positively impacting stress. The results of our research indicated that there is a positive and significant relationship between stress and family–work conflict. This result supports previous studies (Bashir and Ismail Ramay, 2010; Jalagat, 2017; Soomro et al., 2018; Rezeki et al., 2020) where it was demonstrated that stress and family–work conflict creates negative consequences in the working environment, i.e., lower job performance, commitment, and higher turnover intention. In line with a study by Nora and Fitri Anggraeni (2020), our study indicated that family to work conflict is associated with stress. Our research also confirms the relationship in stress and work- family conflict in line with the Smith et al. (2018). Our study found that stress exerts a mediator role between the two conflicts, which is consistent with other studies (Rubio et al., 2015; Ismail and Gali, 2017; Raja et al., 2018; Dodanwala and San Santoso, 2021; Irawanto et al. 2021). These findings confirm the spillover effect of work-family/family–work conflict and stress. On the other hand, we found that the five sectors are significantly different regarding the work-family/family–work conflict and stress. The results showed that retail store employees experienced higher work–family conflict than educational, textile, hospitals, and banks. In addition, all five groups are significantly different based on stress. Results indicated that employees in hospitals experience higher stress than in retail stores, banks, education, and textile employees. Nurses and doctors experience high levels of job stress due to daily exposure to the suffering and anguish of patients in the hospital. Due to the hygienic nature of the COVID-19 pandemic, healthcare professionals have been on the front lines of combating the epidemic. Despite their professionalism, overburdened, overworked, and underequipped healthcare systems may contribute to elevated stress levels (Khan et al., 2021). Medical professionals who have come into contact with any confirmed or suspected coronavirus cases are at risk for psychological and physical health problems, such as stress. Since the outbreak of COVID-19, healthcare workers have been subjected to increased physical and mental stress, including an increased risk of illness, confinement, insufficient protective equipment, exhaustion, and a lack of contact with loved ones. The urgency of the issue is causing medical workers to have more mental health problems (Khan et al., 2021), which affects their capacity to make decisions and might hurt their well-being in the long run (Zeb et al., 2021). Therefore, hospital employees, such as physicians and nurses, are more likely than the general population to develop depressive disorders, such as those caused by job stress. Finally, results showed that all the five groups are also significantly different based on the family to work conflict. Results indicated that the textile group faces a higher level of family–work conflict than bank, hospital, retail stores, and education employees.

### Theoretical contributions

Our study contributes to the literature in several ways. First, this is the first study that examined the spillover effect of work–family conflict, stress, and family–work conflict in the five sectors of Asia during Covid-19 uncertainty. Even though prior studies examined work-family/ family–work conflict as a cause of stress among workers (Bashir and Ismail Ramay, 2010; Jalagat, 2017; Soomro et al., 2018; Nora and Fitri Anggraeni, 2020; Rezeki et al., 2020), the spillover effect of work-family / family-work and stress has been overlooked in the literature as per our knowledge. Therefore, our study observed the influence of work-family / family–work conflict on stress and the impact of stress on these two conflicts to fill this void in the five sectors of the Asian context. Secondly, our study contributed to the stress literature by examining its intervening role between work–family conflict and family–work conflict; also, between family-work and work–family conflict. Earlier studies examined the mediation effect of stress (Rubio et al., 2015; Ismail and Gali, 2017; Raja et al., 2018), however, the mediation of stress specifically between work-family and family–work conflict and vice versa has not been investigated. Finally, our research extended the work-family/ family-work and stress literature by determining that all these sectors (education, textiles, hospitals, banks, and retail stores) are different in experiencing work-family/family–work conflict and stress.

### Managerial implications

Our study suggests the spillover effect of work–family conflict, stress, and family–work conflict in the five major sectors. This idea provides key contributions since theoretical gaps have been filled and organizational psychology research has been extended. This research is significant because it departs from previous studies that explored the impact of work–family conflict on employee stress rather than focusing on the investigation of spillover effects. The study’s novel findings have practical implications for managers and leaders, indicating that managers should inspire and cultivate a healthy-friendly workplace that will drive people to use their energy and strive for success, which would, in turn, reduce work-family / family–work conflict and stress among employees in the working environment. Emphasizing on the fact that pleasant working conditions encourage people to reduce their stress, it is known that employees in an influential organizational culture have fewer work–family conflicts and are more productive (Bakker and Schaufeli, 2008). The work–family conflict is much less frequent in these kinds of workplace cultures. Therefore, organizations have to create an influential culture in their working environment. Organizations should implement and develop measures to avoid or reduce the incidence of work–family conflict and improve work-life balance. In this regard, organizations should think about implementing a flexible timetable and reducing job intensity, among other measurements. In addition, adopting innovative communication solutions based on cutting-edge technology could significantly reduce employee stress (Cascio and Montealegre, 2016).

Furthermore, workers must receive adequate training so as to become aware of work–family conflict and how to mitigate its impact on family life (Lambert et al., 2013). Workers who receive adequate and proper training have less stress at work (Lambert et al., 2009). Workers who already have had the training are more confident at work and could perform their jobs more efficiently, which leads to fewer problems at work and at home. Training and orientation can also help employees to adjust to their new jobs and reduce the shock they feel. In addition, training must be ongoing, and employees must be allowed to submit responses to ensure that it meets the needs of employees (Lambert et al., 2009). Management should hold counseling sessions to learn more regarding the severity of Covid-19, work-family / family–work conflict, and stress among its female employees. These factors should also be addressed by re-designing roles, working shifts, and providing breaks in working hours so they do not interfere with family responsibilities. The administration may organize teams that would provide collegial support and allow employees to express their emotions and issues to overcome them and avoid stress. Emotional intelligence training should be offered in order to improve the ability to control emotions so that emotions become an asset for employees and help them in overcoming the negative consequences of stress rather than exacerbating them (Sharma et al., 2016).

### Study limitations and direction for future research

There are some limitations to the present research, which could lead to new suggestions for future work. First, the quantitative methodologies used in this study may not have captured all of the workers’ perspectives on some issues. Future studies with in-depth interviews may be able to fill this void. Second, the study variables, such as work–family conflict, family–work conflict, and stress were assessed by a self-report technique which might lead to common method variance and consistency bias. Alternative data collection approaches, such as focus groups, could be used in future studies. Because this study is cross-sectional, it is challenging to find causal relationships between variables. As a result, future researchers may also choose to concentrate on longitudinal research. In this research, we have not examined the association of demographics on stress and work/family conflict. Future studies may explore the relationship between gender, the number of kids, and education on these study variables.

## Conclusion

Employees in different professions have to perform their jobs under continuous pressure, currently increased by the pandemic situation. Besides, due to the mismatched natures of work, high job demands, and family responsibilities, they often experience work-family/family–work conflict and stress. The earlier findings of the previous studies indicated work–family conflict has effects on stress. Researchers need to understand the spillover effect of work–family conflict, stress, and family–work conflict. Therefore, our study examined this effect. This research indicated that work–family conflict influences stress, which in turn positively impacts family–work conflict. Our study findings also confirmed that family–work conflict influences stress, which in turn influences work–family conflict positively and significantly. Stress mediates the work–family conflict and family–work conflict relationship and vice versa.

In addition, we found that the five sectors are significantly different regarding the work-family and family–work conflict and stress. Work–family conflict is a critical area of concern for researchers and professionals, as evidenced by the rising body of knowledge in organizational psychology. Our study suggested that if management wants to meet its objectives and reduce employee stress, then there is no better alternative than focusing on the work and personal life of their employees. Management policies should place a greater emphasis on human concerns such as time flexibility, allowing employees to work from home during a family crisis, and establishing a family-friendly work environment to address work-family / family-work difficulties and stress. Support from managers has to be more effective than the organizations or supervisory support in reducing work–family conflict dimensions and stress since management policy and decision-making have a role in decreasing work–family conflict. In the Pakistani working environment specifically, management or policymakers should take a more constructive approach to work-family issues.

## Data availability statement

The datasets generated and analyzed in the current study are available from the corresponding author on reasonable request.

## Ethics statement

The protocol was approved by the Ethics Committee of the Superior University, Lahore, Pakistan. Informed consent was obtained from all individuals included in this study.

## Author contributions

Conceptualization and formal analysis: NE and GA. Data curation: GA. Investigation: IF, GA, and FC. Methodology: GA and FC. Project administration: NE. Resources: IF and GA. Writing: NE, GA, FC, and IF. Review, proof reading and editing: NE, GA, FC, and IF. All authors have read and agreed to the published version of the manuscript.

## Acknowledgments

We thank the Universidad de Málaga, Spain, for the financial support for the publication of this article.

## Conflict of interest

The authors declare that the research was conducted in the absence of any commercial or financial relationships that could be construed as a potential conflict of interest.

## Publisher’s note

All claims expressed in this article are solely those of the authors and do not necessarily represent those of their affiliated organizations, or those of the publisher, the editors and the reviewers. Any product that may be evaluated in this article, or claim that may be made by its manufacturer, is not guaranteed or endorsed by the publisher.
